# Coronavirus Replication: Genomes, Subgenomic RNAs, and Defective Viral Genomes

**DOI:** 10.3390/v17060767

**Published:** 2025-05-28

**Authors:** Rory Williams, Jack Hales, William Collier, Phillip Gould

**Affiliations:** 1 Centre for Health and Life Sciences, Coventry University, Coventry CV1 2UD, UK; ac2921@coventry.ac.uk; 2 OVO Biomanufacturing Ltd., Friars House, Manor House Drive, Coventry CV1 2TE, UK; j.hales@ovobiomf.com (J.H.); w.collier@ovobiomf.com (W.C.); 3 College of Engineering, Environment and Science, Coventry University, Coventry CV1 2UD, UK

**Keywords:** coronaviruses, defective viral genomes, DVGs, recombination, SARS-CoV-2

## Abstract

With the emergence of the SARS-CoV-2 pandemic the process of coronavirus replication has been under increasing scrutiny. During the replication of their genomic RNA, coronaviruses produce a range of other RNAs in addition to the negative-sense replicative intermediates of the genome, which includes a set of subgenomic RNAs. These subgenomic RNAs are nested within the sequence of the complete genome and can be both replicated further and act as templates for protein production. Alongside these functional products of discontinuous replication, coronaviruses produce defective viral genomes that can potentially impact both the virus and infected host cells. These interactions can arise from the ability of these defective viral genomes to impact the production of new infectious virions, through either competition with the wild-type genome for replication or by stimulating an antiviral response. Examining the behaviour of defective viral genomes can also help to elucidate the functional elements of the genome involved in the processes of replication and packaging. This review covers the process of intracellular replication by coronaviruses describing the mechanisms by which the different RNA species are produced. Of particular focus are factors involved in discontinuous replication that produces defective viral genomes, and the behaviour of coronavirus defective viral genomes.

## 1. Human Coronaviruses

As of 2025, the *Coronaviridae* family, belonging to the *Nidovirales* order, has expanded to 54 viral species that infect a range of hosts [1] (pp. 2429–2440). Of these, seven are pathogenic human coronaviruses (HCoVs): HCoV-NL63, HCoV-229E, HCoV-HKU1, HCoV-OC43, Middle Eastern respiratory coronavirus (MERS-CoV), severe acute respiratory coronavirus (SARS-CoV), and severe acute respiratory coronavirus 2 (SARS-CoV-2) [2]. HCoV-NL63 and HCov-229E belong to the α-coronavirus genus, whilst HCoV-HKU1 and HCoV-OC43 belong to the β-coronavirus genus (γ- and δ-coronaviruses make up the other two genera, but no HCoVs fall into either of these categories).

The identity of the historically studied B814 as one of these seven HCoVs or as an additional HCoV is uncertain; as B814 could not be grown outside of human embryonic tracheal organ culture, it was abandoned as a subject of study in favour of isolates that could be cultured in cell lines [3].

Upon infection of humans, four of the seven currently identified HCoVs, -NL63, -229E, -HKU1, and -OC43, cause limited respiratory infections that display symptoms of the common cold [4]. This low severity is not seen in the three other HCoVs that all belong to the β-coronavirus genus: SARS-CoV, MERS-CoV, and SARS-CoV-2.

**Figure 1:**
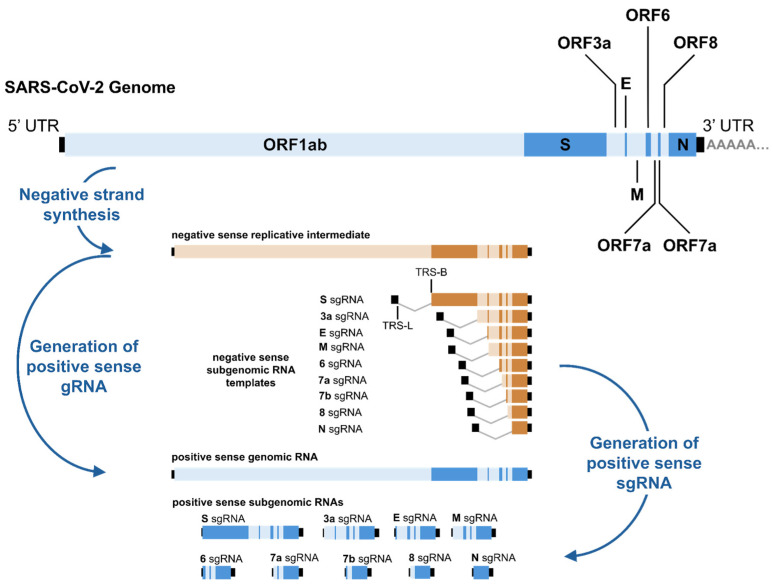
The production of gRNA and sgRNA during SARS-CoV-2 replication cycles. Positive-sense gRNA that has been released into the cell during viral fusion acts as mRNA for the production of pp1a and pp1b, which are subsequently cleaved into nsps that form the viral RTC. Initially, the RTC produces the nested set of sgRNAs during negative-strand synthesis until the DDX1 helicase is recruited to the complex, after which synthesis of a negative-sense replicative intermediate of the genome can occur. A second round of replication then produces a new positive-sense genome that can be packaged into a new viral particle, the structural proteins of which are produced by positive-sense sgRNAs.

Along with their distinctive particle morphology, the *Coronaviradae* share other common features. These viruses contain a large, approximately 30 kb, positive-sense single-stranded RNA genome that replicates through a negative-sense anti-genome intermediate (Figure 1) [5]. Coronaviruses (CoVs) have a conserved genomic organisation: They contain a large (approximately 20 kb or larger) gene encoding the replication/transcription complex (RTC) that occupies the majority of their genomic RNA (gRNA). This replicase gene is composed of two overlapped open reading frames (ORFs): ORF1a and ORF1b, which produce polyprotein 1a (pp1a) and polyprotein 1b (pp1b), respectively, each of which in turn encodes non-structural proteins (nsps) [6]. Alongside their gRNA, coronaviruses, and other members of the *Nidovirales* order, produce subgenomic RNAs (sgRNAs) following cellular entry with these sgRNAs, encoding both structural and accessory proteins.

## 2. Subcellular Location of Coronavirus Replication

**Figure 2:**
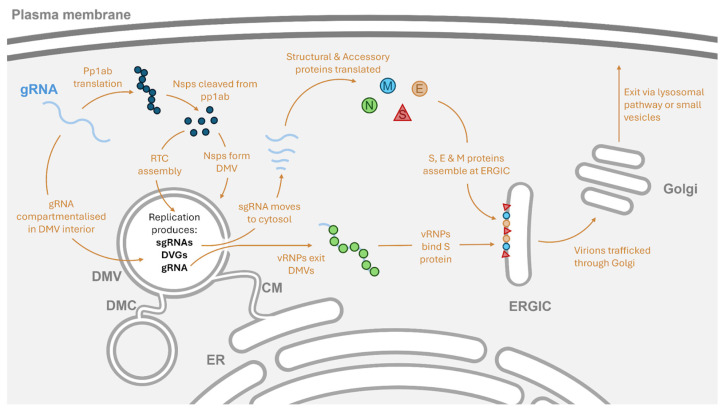
An overview of some of the events that occur during the intracellular portion of the coronavirus lifecycle and their location. Having uncoated in the cytosol, the coronavirus gRNA (light blue, top left) can act as mRNA for the translation of pp1a and pp1b. Cleavage of these polyproteins produces nsps that induce the formation of DMVs, which are connected to the ER by CMs and to other DMVs by DMCs. Having been compartmentalised into the DMVs, gRNA can be replicated by the viral RTC assembled from the nsps. Products of this replication include DVGs, gRNA, and sgRNAs. Through a pore complex in the DMV, sgRNAs can enter the cytosol and be translated into the structural and accessory proteins. Both DVGs and gRNAs can also exit via the pore complex, and once in the cytosol bind N proteins to form vRNPs. These vRNPs can interact with the other structural proteins found in the ERGIC membrane to initiate the assembly of a new viral particle, which is trafficked through the Golgi, wherein structural proteins receive post-translational modifications. Subsequently, the virions exit the cell non-lytically through either the lysosomal exit pathway or secretion in small vesicles.

During cellular infections, HCoVs have been observed producing multiple subcellular structures in the cytoplasm (Figure 2). Rather than directly replicating in the cytoplasm, where the viral RNA would be exposed to the innate immune system, many viruses first form replication organelles to sequester their genome from the intracellular immune system’s receptors, as well as concentrate the resources for nucleic acid synthesis and viral replication [7]. In HCoVs, the structures formed upon cellular infection include double-membrane vesicles (DMVs), the smaller double-membrane spherules (DMSs), and convoluted membranes (CMs) [8]. During CoV infection, CMs, derived from the endo-plasmic reticulum, joined to DMVs appear to form first [9,10], and during MERS-CoV infections, DMVs surrounding CMs can also be observed in the later stages of infection [11]. This has led to the suggestion that the DMVs are formed first during infection, followed by the formation of the CM structures.

Experiments using metabolic labelling of viral RNA to detect newly synthesised viral RNAs, combined with quantitative electron microscopy autoradiography, showed that the DMVs appear to be the site of RNA synthesis across human coronaviruses [8]. RNA synthesis could not be linked to the other structures induced by viral infection such as DMSs or CMs [8]. Despite electron microscopy images of coronavirus replication organelles being generated as early as 2002 [12], these early experiments failed to detect the presence of an opening or pore in the DMVs that would enable the movement of resources into the replication organelle, or the movement of products such as sgRNAs encoding structural or accessory proteins out of the organelle to the host ribosomes.

A transmembrane pore that may be responsible for enabling the movement of resources required to replicate the viral genome into the replication organelle and the movement of viral RNA out of replication organelles has been observed in mouse hepatitis virus (MHV), a non-human CoV from the β-coronavirus genus. In this study, electron tomography was performed on MHV-infected cells, the cryo-lamellae prepared from these MHV infected underwent ion milling, and subsequently, the tomography revealed previously unobserved features: the presence of filaments, assumed to be RNA, in the DMV lumens and the presence of a double-membrane-spanning molecular pore that was shown to contain the conserved nsp3 [13].

The detection of nsp3 as part of a complex led to the suggestion that this pore was responsible for the movement of the viral RNA genome into the cytosol, where nucleocapsid (N) protein could bind the genome, thus initiating the assembly of a novel virion, as conserved domains in the nsp3 protein can bind to both ssRNA and the N protein [14,15]. A second electron tomography study on SARS-CoV-2-infected cell lines also found that SARS-CoV-2 induces the formation of DMVs that contain large amounts of RNA in their lumens [16], and described regions in the DMVs where the inner and outer membranes were clamped together, suggesting that this deformation could be due to the action of the protein pore complex described by Wolff et al. [13].

More recently, in situ cryo-electron tomography on cells prepared with focused ion beam milling showed that transfecting a plasmid expressing nsp3 and nsp4 into the cells triggered the formation of DMVs and led to the formation of the pore complex [17]. Previously unreported double-membrane structures linking the DMVs, termed double-membrane connectors (DMCs) (Figure 2), were also observed to form after the expression of nsp3 and nsp4 [17]. The expression of nsp3 and nsp4 as uncleaved polyproteins was sufficient to trigger the pairing of membranes, but only after the two proteins were cleaved by the Papain-like protease (PL^pro^) did the DMVs and pore complexes form [17]. A subsequent study was then able to reveal the molecular architecture of the pore complex as consisting of 12 copies of each nsp and show that the complex contained a central channel that could enable RNA translocation [18]. Notably, cellular RNA appeared to be excluded from the DMVs containing fully formed pore complexes in cells transfected with nsp3 and nsp4, suggesting that the translocation of RNA by this pore structure may require additional factors [17].

Whilst the initial studies by Wolff et al. and Klein et al. did not report any structures being seen that could have been the replication complexes in the interior of the DMVs [13,16], more recent studies appear to confirm the DMVs as the site of RNA synthesis (Figure 2). The N-terminal domain of nsp3, which is exposed to the lumen of the DMV, can recruit the SARS-CoV-2 RTC to the interior of the DMV membrane via interaction with one of the core components: nsp12 [19]. Nsp6 appears to support the replication organelle by establishing the DMCs that enable the movement of lipid droplets to DMVs, whilst gatekeeping proteins from the DMVs as they change in size during RNA synthesis [19,20].

## 3. The Coronavirus Replication Complex

The 5′ capped and polyA-tailed nature of the positive-sense RNA genome allows for a coronavirus genome to act as mRNA immediately upon cellular entry using host cell ribosomes to produce pp1a and pp1ab. Production of two potential polyproteins, pp1a or the longer pp1ab, which includes the translation of ORF1b, from the overlapping ORFs 1a and 1b is enabled by ribosomal frameshifting [21]. The presence of an RNA pseudoknot and “slippery sequence” leads to a −1 frameshift during the translation of ORF1a. Having shifted out of frame with respect to the ORF1a stop codon the translated sequence extends into the ORF1b, producing the pp1ab [21,22].

Cleavage of these polyproteins is carried out by proteases that are encoded in ORF1a and can act autocatalytically to produce smaller nsps that together form the viral replication and transcription complex (RTC) [6,23,24,25]. In SARS-CoV-2, these cleavage events are performed by the PL^pro^ and main protease (M^pro^) found within nsp3 and nsp5, respectively [26]. Post-cleavage, the nsps are able to form the viral RTC that produces both gRNA and its intermediate negative-sense form, produce sgRNAs during synthesis of the negative-sense gRNA intermediate, remodel the internal structure of the cell to form the replication organelles, and carry out other functions that support viral replication [25].

In total, fourteen of the nsps have had either direct or indirect roles in replication associated with them [25], but nsps 7, 8, and 12 may be the core proteins needed for the synthesis of new RNAs. Nsp8 appears to be capable of binding template RNA and initiating primer-independent synthesis of short sequences but does not appear responsible for the synthesis of new gRNA or sgRNAs [27]. The short RNA sequence produced by nsp8 that is bound to the template strand may act as a primer for the primer-dependent RNA synthesis catalysed by the nsp12 subunit of the viral RNA-dependent RNA polymerase (RdRp) [27]. Nsp7 appears to be an essential co-factor for nsp8, which increases the RNA binding affinity of nsp8; in turn, the nsp7–nsp8 complex then increases the RNA polymerase activity of nsp12 [6,28]. When complexed together, these three nsps are able to extend RNA sequences, and together, nsp7, nsp8, and nsp12 may enable the core functions of the viral RdRp responsible for the synthesis of new viral RNAs [29]. The structure of the SARS-CoV-2 nsp7, nsp8, and nsp12 replication complex on an associated template strand was able to be elucidated following the addition of these three proteins to an RNA template and purification of the resulting RNA–RdRp complexes [29]. Single-particle cryo-EM showed that two turns of duplexed RNA could be seen interacting with the replication complex, which was a greater amount of duplexed RNA associated with the RdRp than in other RNA viruses [29]. This was enabled by the presence of two nsp8 extensions that interacted with duplexed RNA up to 28 nucleotides away from the active site of the replication complex, with this interaction potentially stabilising the RNA–RdRp complex, thus preventing disassociation and enabling the replication of the exceptionally large coronavirus genomes [29].

Having formed the replication complex and replication organelles, the positive-sense genome is either replicated through a negative-sense intermediate to produce new gRNAs or, during the negative-strand synthesis, sgRNA is formed using the conserved leader sequence-to-body sequence template-switching mechanism [30]. Using the SARS-CoV-2 genome as an example, these products of coronavirus replication are displayed in Figure 1.

## 4. The Initiation of Genomic RNA Replication

Following experiments in group 2a CoVs, two key structural elements found within the final 301 nucleotides of the 3′ untranslated region (UTR) of coronaviruses have been implicated in controlling the initiation of negative-strand synthesis. Within this UTR, a bulged stem-loop is found in proximity to the end of the N protein ORF, and closer to the 3′ terminus of the genome another structure in the form of a hairpin stem-loop can be found [31]. Studies in MHV and bovine coronavirus (BCoV) have shown that these two structures are essential for the replication of full-length gRNA and that their presence enables the replication of defective viral genomes (DVGs) in both MHV and BCoV [31,32,33,34]. The folding of this 3′ UTR region can adopt two mutually exclusive topologies with the complex pseudoknot topology formed by the interaction of a region at the base of the bulged stem-loop and the loop portion of the downstream hairpin stem-loop [35]. This topology does not appear to be the default topology of the region: formation of this pseudoknot structure involving the bulged stem-loop and upstream hairpin stem-loop is prevented by an interaction between the hairpin stem-loop and the extreme end of the genome [35].

Based on the extensive examination of the two structures in the 3′ UTR region in MHV, it was proposed that these two mutually exclusive topologies form a molecular switch that regulates viral RNA synthesis [31,32].

Following this proposal, Züst et al. were able to demonstrate interactions between nsp8 and nsp9 with loop 1 of the pseudoknot, as well as interactions between this same section of the pseudoknot and the 3′ terminus of the genome that is preventing the topological change [35]. This led to the proposal of a model of negative-strand synthesis initiation by the researchers: The nsp8 primase, in a complex with other nsps, binds to the 3′ end of the genome, which is base-paired to the pseudoknot loop 1 region. Following this, the primase activity of nsp8 synthesises a short strand of negative-sense RNA that separates the 3′ terminus and the pseudoknot loop 1 sequence from each other. Having done so, the base of the bulged stem-loop and the loop region of the adjacent hairpin stem-loop are no longer prevented from interacting; subsequently, this interaction alters the topology of the region by forming the pseudoknot structure in the 3′ UTR. The newly formed pseudoknot structure then provides an assembly site for the RTC, and nsp12 can subsequently elongate the negative strand from the short primer sequence already present due to the action of the nsp8 primase [35].

The synthesis of positive-sense RNA assumedly starts at the 5′ UTR of either the coronaviral gRNA or sgRNA. Given that sgRNA synthesis takes place during the production of negative-sense RNAs [30], negative-sense sgRNA must contain the minimal requirements to be replicated back into protein-coding positive-sense RNA.

The 5′ UTR of coronavirus genomes contains multiple stem-loop structures with five stem-loops predicted to be conserved across human and animal coronaviruses, with different subgroups having high levels of similarity in their 5′ UTR structures [36]. Across the different coronavirus groups, stem-loops 1 and 2 occur upstream of stem-loop 3, which contains the core sequence of the transcriptional regulatory site (TRS) leader (TRS-L) [36]. This means that all coronavirus gRNAs and sgRNAs will contain these structures, with the base region of stem-loop 1 and the loop portion of stem-loop 2 being suggested as important for enabling replication [37,38]. Experiments mutating the structure of stem-loops 1, 2, and 4 in BCoV demonstrated that their disruption could affect the replication of a DVG-like RNA construct containing the 5′ UTR [39,40]. Given that stem-loop 4 cannot be a strict requirement for replication, as it will be lost in all sgRNA, it may function as an element enhancing the replication of the full-length gRNA. A role for the fifth stem-loop in replication has not been described, and this stem-loop, absent in sgRNA, has been suggested to play a role in the packaging of viral RNA instead [41].

## 5. The Synthesis of Genomic RNA and Subgenomic RNA

Coronaviruses produce multiple short RNA sequences that encode the viral structural and accessory proteins, with these shorter sgRNA sequences “nested” within the full-length genome. These sgRNAs are formed through a discontinuous replication event in which the synthesis of nascent negative-sense RNA strands is paused and the nascent strand is transferred from one site in the genomic template to another (Figure 2). This process is mediated by the TRSs in a process conserved across *Nidovirales* genomes and coronaviruses by extension [42].

A TRS-L site is present at the 5′ end of the positive-sense genome. After ORF1a and ORF1b, upstream of each of the structural or accessory proteins in the viral genome a TRS-Body (TRS-B) sequence is present. The TRS-B sequences contain a core sequence that has an identical nucleotide sequence to the core sequence of the TRS-Leader [43]. As the nascent strand synthesis begins from the 3′ end of the genome, moving over the structural proteins and associated TRS-B sequence, the synthesis can halt. The mechanism through which nascent strand synthesis is paused has not been determined, but secondary structures proximal to the body sequences and the action of viral proteins have been hypothesised as playing a role [44].

**Figure 3:**
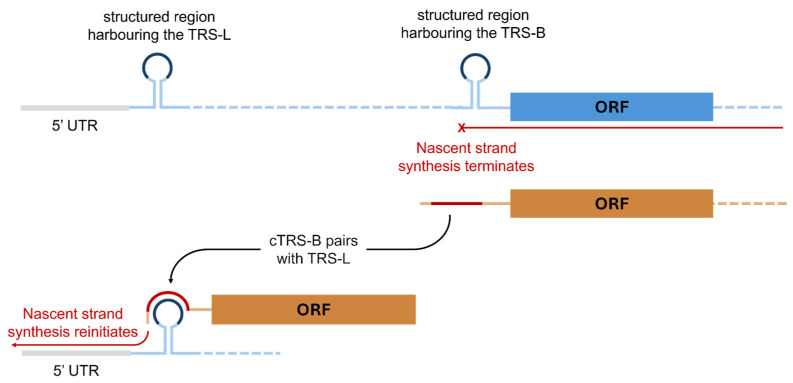
During negative-strand synthesis, the TRS-B is copied into the nascent strand, forming a cTRS-B sequence. After the termination of nascent strand synthesis, the core sequence of the TRS-L (dark blue) and complementary core sequence in the cTRS-B (dark red) can base-pair. This might enable the reannealing of the nascent strand and template strand, enabling RNA synthesis to restart. The TRS sequences are found within regions containing RNA secondary structures, which are represented in this figure by simple stem-loops.

Whatever the mechanism may be, after attenuation of nascent strand synthesis the TRS-B sequence that has been copied into the nascent strand (cTRS-B) is able to base-pair with the distal TRS-L sequence in the genomic template (Figure 3). When adding a novel core sequence that matched the core sequence of the native TRS-Leader in transmissible gastroenteritis virus (TGEV), a pig coronavirus, it was observed that core sequence complementarity alone could not generate detectable sgRNA formation [43]. Alongside the base pairing of the core sequences in the two strands, additional nucleotides in the cTRS-B sequence can base-pair with the TRS-L, and this extra base pairing outside of the complementary core sequences was required for sgRNA generation [43].

After the nascent strand forms the cTRS-B-to-TRS-L interaction, synthesis of the negative-sense nascent strand can reinitiate (Figure 3). This process produces short sgRNAs that contain one or more ORFs from the 3′ end of the genome and a short subsection of the 5′ UTR upstream of the TRS-L. As they maintain the terminal sequences, these short negative-sense sgRNA sequences can be recognised by the viral RdRp for use as a template. This produces positive-sense sgRNA that can subsequently act as mRNA templates for the translation of viral structural proteins [45].

The interaction between the TRS-L and cTRS-B on the nascent RNA may be facilitated by long-range interactions between RNA sequences in the coronavirus genome that bring the TRS-L and TRS-B sites on the template genome into close spatial proximity despite their genomic distance, so when the nascent strand synthesis terminates the nascent strand is already in close proximity to the TRS-L sequence on the template [45]. The first evidence for the importance of these long-range RNA–RNA interactions within the viral genome was from studies on TGEV. In this coronavirus, the interaction of two RNA sequences located at either end of the genome and separated by more than 25,000 nucleotides was required for the formation of sgRNA encoding the N protein [46,47]. More recently, Ziv et al. used crosslinking of matched RNAs and deep sequencing (COMRADES) to map the sites of RNA–RNA interaction within the SARS-CoV-2 genome and found long-range interactions between the 3′ and 5′ extremes of the genome [48]. Nucleotides around position 80 in the SARS-CoV-2 genome were able to interact with nucleotides around position 29,847, which causes the genome to adopt a cyclic conformation where the ends of the genome are in close spatial proximity [48]. A distance of over 29.7 kb between the interacting nucleotides marks this RNA–RNA interaction discovered by Ziv et al. as “among the longest distance RNA-RNA interactions ever reported” [48].

The phosphorylation of the N protein by a host cell kinase enables the recruitment of the host DDX1 RNA helicase to the RTC [49]. The helicase then enables the synthesis of full-length gRNA intermediates and the longer set of sgRNAs, as well as a reduction in the synthesis of the shorter sgRNAs, providing a mechanism by which CoVs can balance sgRNA and gRNA synthesis [49]. The DDX1 helicase’s recruitment to the RTC allows the viral replication machinery to efficiently read through the secondary structures on the gRNA template, enabling the production of a full-length negative-sense intermediate that subsequently acts as a template for full-length positive-sense gRNA, or to progress further through the genome to reach sgRNA regions deeper in the genome prior to replicative stalling and the process of sgRNA formation [49].

Quantification of the relative abundances of the gRNA and sgRNAs 12, 14, and 24 h post-infection revealed that the majority of mRNAs purified from SARS-CoV-1- or SARS-CoV-2-infected Vero E6 cells were sgRNAs, with genomic RNA accounting for 6% and 5.1% of the purified RNA from SARS-CoV-1- and SARS-CoV-2-infected cells, respectively [50]. The relative quantities of sgRNAs to the genome differed between the two tested viruses, but sgRNA encoding the N protein was the most abundant RNA in both cases [50]. In addition to the core sequence of the TRS-L and TRS-B sites, additional nucleotides around the TRS-B site can contribute to the strength of the interactions between the TRS-L and the cTRS-B in the nascent strand [51]. The differing minimum free energy required to form the cTRS-B-TRS-L interaction between the nascent and template strand appears to correlate with the detected levels of SARS-CoV-2 sgRNAs in cell culture, suggesting that modulating the relative strength of the different RNA–RNA interactions between the two strands by decreasing the energetic requirements to form the interaction acts as a mechanism that helps determine the relative quantities of the sgRNAs formed [51]. However, this may not be the only mechanism influencing the differing quantities of the sgRNAs produced during replication. Rearranging the order of the nested sgRNA regions in the gRNA of MHV whilst maintaining the TRS-B sequence and nucleotides surrounding the site led to increases in the production of sgRNA the closer it was positioned relative to the 3′ end of the gRNA [52]. Likewise, increasing distance from the 3′ end of the genome decreased the quantity of sgRNA that was synthesised [52]. Increasing the amount of secondary structure the replication complex is required to navigate before it can synthesise a given sgRNA by moving the region containing the sgRNA away from the 3′ end of the genome may increase the probability of stalling and detachment before the sgRNA can be successfully produced. This would be in line with the finding that DDX1 helicase recruitment is required for synthesis of sgRNAs further from the 3′ end of the genome and synthesis of full-length gRNA [49].

The number of structural proteins and associated sgRNAs produced by each HCoV varies, but they all produce four essential structural proteins from sgRNA templates in order to enable assembly of new viral particles: the spike (S), envelope (E), membrane (M), and N proteins, with group 2a coronaviruses such as OC43, BCoV, and HKU1 producing a fifth protein, the haemagglutinin esterase (HE) protein [53].

HCoVs are able to methylate the 5′ end of their RNAs through the concerted actions of nsp10, nsp13, nsp14, and nsp16. This enables recruitment of the host’s translation factor eif4E, which allows for binding of the virally produced mRNAs to ribosomes for translation [54]. In addition to a 5′ cap, the gRNA and sgRNAs produced by coronaviruses are polyadenylated [55,56]. The 3′ UTR of SARS-CoV-2 is missing a canonical polyadenylation signal, meaning that host polyadenylation machinery is not likely to be responsible for the polyA tail on the viral RNAs [56]. Rather, it is suggested that nsp8, which has been shown to act as an adenylyltransferase in HCoV-229E, is carrying out a conserved function in SARS-CoV-2 [56,57].

A study using BCoV as a model coronavirus found that the length of the polyA tail on both gRNA and sgRNA varies across the duration of cell culture infection, increasing from approximately 45 nucleotides in length immediately following infection to approximately 65 nucleotides between 6 to 9 h post-infection before subsequently decreasing to approximately 30 nucleotides at the much later timepoint of 120 h post-infection [55]. PolyA tail length appeared to enhance RNA translation, and it was suggested that the varying polyA tail length may provide a mechanism by which coronaviruses can dictate whether a gRNA or sgRNA is to be used for translation or replication [55]. A model explaining how coronaviruses might extend their polyA tails has been proposed by Peng et al. [58]: During negative-strand synthesis, the length of the polyU tail on the nascent strand is dictated by the length of the polyA tail on the template strand. However, during synthesis of the positive-sense strand from this negative-sense template, an AGUAAA hexamer in the positive strand acts as a site for the binding of host factors that promotes polyadenylation, forming a stable RNA–protein complex capable of extending the polyA tail being synthesised beyond the length of the polyU tail on the negative-sense template [58]. How the length of polyA tail decreases was not described, but it may be possible that instead of losing the polyA tails, the fraction of gRNA with shorter tails increases late into the infection, as these RNAs may not be directed to translational machinery and are instead used to produce new gRNA and sgRNAs.

## 6. Defective Viral Genomes

DVGs are considered to arise from recombination events during viral genome replication, leading to the loss of some, or all, essential protein-coding regions, but the retention of genomic elements responsible for enabling further replication and packaging of the genomes [59]. Because these genomes have maintained their ability to be replicated and packaged in competition with the standard viral genome, consuming resources essential for the production of new virions and yet not producing any of these resources themselves, they are able to interfere with the production of infectious viral particles [60]. Since their initial discovery in influenza, DVGs have been detected in a wide range of viruses, and their ability to interfere in viral replication has led to suggesting them as a novel class of antiviral therapeutic [59].

**Figure 4:**
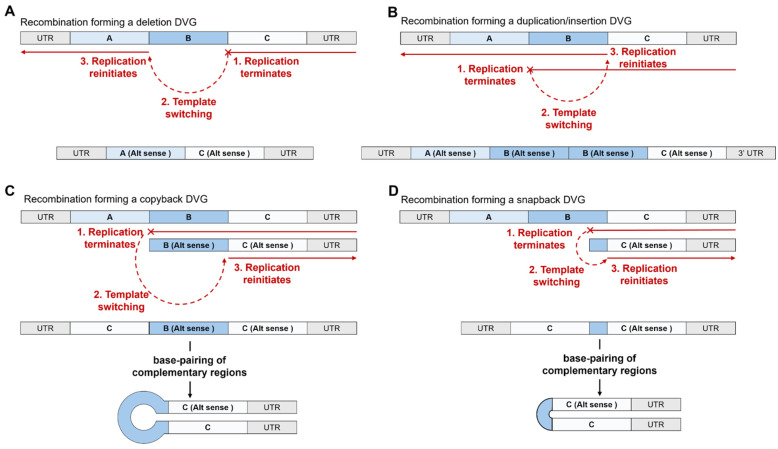
Types of defective viral genomes described in coronaviruses. (**A**) Deletion DVGs arise when the replication complex terminates replication (1) and undergoes template-switching to a region downstream of the site of detachment, “skipping” a region of the genome (2), before re-initiating replication at this downstream location (3), leading to the deletion of genomic content in the resultant genome. (**B**) Duplication/insertion DVGs arise when the replication complex terminates replication (1) and undergoes template-switching to a region upstream of the site of detachment (2) before re-initiating replication at this upstream site (3), “duplicating” a region of the genome or “inserting” extra genomic content (B’) into the newly synthesised genome. (**C**) Copyback genomes are formed when the replication complex terminates replication on the template strand (1) before template-switching to the nascent strand (2) and re-initiating replication on the nascent strand, “copying back” over a region of already-replicated sequence (3). The resulting genome contains two regions that are complementary, and these regions undergo base-pairing, forming a hairpin-loop structure if there is a long region of genome between the two complementary sections. (**D**) If there is only a short region of genomic sequence between the two complementary sections of a copyback DVG, then the loop structure is absent and the genome is sometimes referred to as a “snapback” DVG. Copyback/snapback DVGs can form during either positive- or negative-strand synthesis, leading to genomes with two copies of either the 3′ UTR (with one copy inverted) or two copies of the 5′ UTR (with one copy inverted).

Vignuzzi and López provided a review of the viruses in which DVGs were described as of 2019 [60], illustrating the range of viruses that produce DVGs, with DVGs observed in examples of positive-sense RNA viruses, negative-sense viruses, and double-stranded RNA viruses, and in human immunodeficiency virus 1 (HIV-1) as the only example of a retrovirus that can produce DVGs identified to date [60]. Some of the commonly observed types of DVGs relevant to coronaviruses that have been described in the literature are shown in Figure 4.

Absent from this figure are the more complex intermolecular recombination events, such as those between the different genomic segments of viruses with segmented genomes, as observed in flock house virus and equine influenza virus [61,62]. However, this is not to imply that such recombination events do not occur in coronaviruses; rather, phylogenetic analysis has previously suggested that the HE protein unique to group 2a coronaviruses has a common origin to the influenza C HE protein, with one potential explanation for the common origin being that the gene was transferred to an ancestral coronavirus genome via heterologous recombination during coinfections [63,64].

Some research has used the terminology “structural variants” to describe genomic alterations of 50 base pairs or more, including those arising from deletion of content and duplication of content [65], and this terminology has been used in literature concerning viruses such as SARS-CoV-2 [66]. To avoid confusion, it may help to consider DVGs products of structural variation, or a type of structural variant with specific behaviours (i.e., able to be replicated with the aid of the wild-type genome, but unable to carry out a productive viral life cycle on their own) arising from recombination events in viruses. During this review, the term DVG is used rather than structural variant, but the reader should be aware that this term exists with an overlapping definition. Furthermore, no distinction is made between copyback and snapback DVGs, with the term “copyback” used in this review to include both types of DVG. Figure 4 only displays single recombination events; as discussed below, there is evidence that coronaviruses can produce DVGs harbouring multiple recombination events.

The renewed interest in coronaviruses following the emergence of SARS-CoV-2, the potential therapeutic applications of DVGs [59,67,68,69,70], and the development of multiple next-generation sequencing (NGS) analysis tools for detecting DVGs [71,72,73,74,75,76,77] has led to an increase in the research surrounding coronavirus DVGs. Studying the mechanisms behind the formation of DVGs may help develop an understanding of sgRNA and gRNA replication. In particular, examining the deletion DVGs that are propagated by the virus can help elucidate the minimal requirements for replication and packaging.

### 6.1. The Types of Defective Viral Genome Observed in Coronaviruses

Historically, the presence of deletion DVGs has been specifically studied, with the detection of such genomes reported in multiple coronaviruses: Berne virus, BCoV, infectious bronchitis virus (IBV), MHV, TGEV, and SARS-CoV-2 were all seen to produce deletion DVGs [78,79,80,81,82,83,84]. More recently the NGS analysis software DVGfinder (version 3.1) has been able to detect and characterise recombinant junctions in RNA sequencing data derived from OC43 and MHV stocks, leading to the observation of recombinant junctions that would form deletion, duplication, and both types of copyback DVGs [85], suggesting that coronaviruses may form the complete range of DVGs. In RNA sequencing datasets from SARS-CoV-2-infected cell lines and patient nasal swabs, the presence of deletion DVGs has been detected using another NGS analysis tool that detects DVGs: Viral Recombination Mapper (ViReMa) [83,86].

A diverse range of deletion DVGs was observed in both in SARS-CoV-2 and MERS-CoV illumina sequencing datasets using ViReMa, with the presence of the DVGs validated using nanopore direct RNA sequencing [86]. After normalising the number of detected junctions against reads aligning to the reference genome, it was observed that despite the identical growth conditions, SARS-CoV-2 generated over 10-fold more deletion DVG junctions than MERS-CoV, and when comparing just the number of unique deletion DVGs, SARS-CoV-2 generated 2.5-fold more unique deletion DVGs, suggesting that coronaviruses may produce DVGs at different rates [86]. SARS-CoV-2 also generated more sgRNA than MERS-CoV alongside the increased number of unique deletion DVGs, which may suggest that SARS-CoV-2 is more predisposed towards discontinuous replication events than MERS-CoV.

Nanopore sequencing of deletion DVGs in SARS-CoV-2 has enabled the observation of genomes that contain two internal deletion events on either side of the region encoding nsp12 [84], suggesting that the large human coronavirus genomes can produce DVGs that harbour multiple recombination events, as has previously been observed in equine arterivirus [87].

### 6.2. The Impact of Coronaviral DVGs on Coronavirus Replication and Hosts

When SARS-CoV-2 was grown in primary human lung epithelial (PHLE) cells, some samples contained an increased number of unique DVGs and total DVGs, with these two measurements positively correlating in these stocks from the PHLE cells as well as stocks produced in Vero E6 cells [83]. Comparing the high DVG samples from PHLE cells with the remaining samples revealed the upregulation of genes associated with the antiviral type I interferon (IFN) response, suggesting that SARS-CoV-2 DVGs can trigger IFN responses [83]. Single-cell RNA sequencing further confirmed that, in infected PHLE cells harbouring one or more deletion DVGs, IFN expression was accelerated relative to infected cells without deletion DVGs and that 2 days post-infection, the expression of ISGs was enhanced relative to the DVG-less cells [83].

In clinical patient data, SARS-CoV-2 genomic read counts were lower in asymptomatic patients, and the symptomatic patients who had higher numbers of SARS-CoV-2-associated reads also had higher proportions of unique deletion DVGs and deletion DVG-containing reads [83]. This raised an interesting link between the presence of SARS-CoV-2 DVGs and the development of symptoms during an infection. Deletion DVGs were seen to enhance IFN responses, and it is known that dysregulated overexpression of cytokines is related to the severity of SARS-CoV-2 infections. However, the timing of the response has been seen as critical, with strong and early responses being beneficial for viral clearance [88,89]. Zhou et al. observed that cells harbouring deletion DVGs developed an IFN response faster than infected cells without DVGs [83], suggesting they may enable an earlier antiviral response by infected cells.

It may be possible that in symptomatic patients there is a higher level of viral replication, leading to more viral particle production and the development of symptoms, but with the higher level of SARS-CoV-2 replication, DVG synthesis increases. When comparing symptomatic patients, separating this population into patients with increased disease severity and those with lower disease severity might help determine whether differences in deletion DVG levels and associated IFN induction are affecting the severity of the disease or whether deletion DVG levels are acting as a biomarker of SARS-CoV-2 replication in the patients.

In a single persistently infected patient, sine-wave fluctuation in the proportion of SARS-CoV-2-aligned reads that contained deletion DVG junctions was observed [83], mirroring the behaviour of DVGs in influenza A virus (IAV). This fluctuation arises from the auto-interference of the DVGs in their own replication as they inhibit the production of infectious viral particles [90,91]. If SARS-CoV-2 deletion DVGs, like other CoV deletion DVGs, can exert an inhibitory effect on the replication of SARS-CoV-2 virions, then it would be surprising that increased levels of DVGs would lead to increased disease severity, instead of serving to reduce severity by slowing the spread of the virus.

Deletion DVGs that were constructed through targeted removal of sections of the SARS-CoV-2 genome reduced SARS-CoV-2 titres by 1–2 log10 PFU/mL when transfected into Vero E6 cells and by 2–3 log10 PFU/mL when transfected into Syrian golden hamsters prior to SARS-CoV-2 infection in comparison to a control RNA sequence [67], supporting the idea that deletion DVGs might reduce SARS-CoV-2′s severity. Furthermore, when testing the effects of a poliovirus deletion DVG on SARS-CoV-2-infected mice, Xiao et al. found that treatment pre- and post-infection would reduce the titre of SARS-CoV-2 in the lung tissues by 1.5–3 log10 PFU per gram of lung tissue, and reported that in both infected and uninfected mice treated with the deletion DVG RNA, “there was no weight loss or signs of distress” despite the DVG acting by inducing a strong type I IFN antiviral response [69]. Furthermore, treating mice with this immunostimulatory poliovirus deletion DVG was seen to reduce the histopathological changes to lung tissue associated with SAR-CoV-2 infection [69].

By comparing their deletion DVG constructs to a non-viral luciferase-encoding RNA that provoked similar cytokine responses to the synthetic DVGs, Chaturvedi et al. showed that replication competent RNA sequences derived from SARS-CoV-2 exert a greater inhibitory effect on the virus’s replication [67]. When comparing two SARS-CoV-2 deletion DVGs designed by the deliberate removal of regions of the genome, Yao et al. found that only the DVG that could be replicated by SARS-CoV-2 exerted an inhibitory effect on SARS-CoV-2 replication, whilst the other DVG failed to reduce viral titres [70]. Therefore, whilst innate immune stimulation by DVGs can play a role in reducing viral titres, it appears that replicative interference by a DVG against its parental virus enhances the inhibitory effect.

During serial passaging at a consistent multiplicity of infection (MOI), the accumulation of deletion DVGs coincided with a decrease in viral titre, with the final passage producing a viral titre 55-fold lower than that produced during the first passage prior to the accumulation of DVGs in the stock [84]. Using in vitro transcription to reproduce specific deletion DVGs observed during this serial passaging enabled their transfection into SARS-CoV-2-infected Vero E6 cells, which confirmed that the addition of the deletion DVG would reduce viral titres by at least 1 log10 PFU/mL [84]. During the course of 30 serial passages in Vero E6 cells, a single DVG reached a high frequency within the DVG population, accounting for over 80% of the detected DVGs, and remained dominant between passages 20 and 30 (where the experiment ended), suggesting that some deletion DVGs produced by SARS-CoV-2 may have high replicative fitness [84]. This replicative fitness would be essential for mediating replicative interference. However, during a second series of serial passages, the highest frequency deletion only accounted for 25% of the population at its peak frequency, suggesting that a population dominated by a single deletion DVG may not always arise during SARS-CoV-2 infections [84].

Whilst the effects of duplication/insertion DVGs as well as both types of copyback DVG remain unclear in coronavirus infections, the accumulating evidence suggests that the deletion DVGs are able to exert inhibitory effects on coronavirus replication through the combined mechanisms of innate immune stimulation and replicative interference.

Much of the research on DVGs has focused on genomes that have lost the ability to produce proteins, but in coronaviruses, DVGs can be used as templates for the production of sgRNAs if they retain the TRS-L site in the 5′ UTR and one or more TRS-B sites [84,92]. The difference in possible effects that a DVG with sgRNA-producing capacity may have on the replication of the virus is not known. It may be possible that by producing the structural proteins found within sgRNAs these DVGs can aid the parental virus in creating new virions, furthering their own propagation.

Some of the DVGs observed by Girgis et al. were capable of producing a protein from the regions of nsp1 and nsp10 that were concatenated during the recombination events [84]. The ectopic expression of this protein in SARS-CoV-2-infected cells reduced viral titres, suggesting that the protein may exert an inhibitory effect on the virus [84]. A similar phenomenon has been observed in IAV, wherein a truncated PB protein that forms part of the influenza RdRp can interfere with viral replication [93].

The effects of the different types of DVG produced by coronaviruses have not yet been examined in depth. Thus far, only the effects of deletion DVGs have been studied. Their interference in coronavirus replication has been observed in multiple studies, as has their immunostimulatory nature [67,69,70,84]. This inhibitory effect has led to deletion DVGs being examined as a possible class of therapeutics for treating SARS-CoV-2 infections [67,68,69,70]. The observation that the presence of deletions DVGs in PHLE cells both enhanced and accelerated antiviral responses led Zhou et al. to conclude that DVGs may modulate host responses and affect disease outcomes [83]. Whether the other types of DVGs can mediate similar effects to the deletion DVGs is unknown, and how the natural generation of the different types of DVGs might impact viral pathogenesis requires further study.

### 6.3. Mechanisms and Factors Underlying the Formation of DVGs in Coronaviruses

When examining the deletion DVGs arising from cell culture infections, Zhou et al. observed hotspots for detachment and re-initiation of replication and, through PCR amplification of deletion genomes followed by sanger sequencing, verified the existence of deletions formed through recombination between these hotspots [83]. During further experiments, SARS-CoV-2 was grown in primary lung epithelial cells isolated from donors of different ages. Across the different samples, the researchers re-observed the same hotspots for recombination in the separate populations of DVGs that arose from the infections [83]. Hotspots for recombination events have been observed in more CoVs than just SARS-CoV-2. When comparing deletion DVGs in MERS-CoV, SARS-CoV-2, and MHV, recombination hotspots were seen in all three viruses, with the first 8000 nucleotides of the 5′ end of the genome and the final 10,000 nucleotides of the 3′ end of the genome harbouring the majority of the hotspots [86]. This suggests that the formation of deletion DVGs is not a random process, which may not be surprising if the reliably produced set of sgRNAs generated during coronavirus infections are considered a type of deletion recombination event. In line with this suggestion, Girgis et al. observed that of the hotspots for deletion recombination events in SARS-CoV-2 grown in Vero E6 cells, half of the recombination hotspots included a TRS site, suggesting a propensity for detachment or reattachment around these sites [84]. This was also the case in MHV and MERS-CoV [86].

The long genomes of coronaviruses may render them prone to the detachment of the RTC and more likely to undergo discontinuous replication during gRNA synthesis than viruses with shorter genomes. It is possible that coronaviruses have found a way to derive functionality from the unavoidable discontinuous mode of replication, using this phenomenon as a mechanism to produce sgRNAs.

Similarities between the formation of sgRNA and deletion DVGs can be found in the way in which the re-initiation site on the template RNA is determined. It has previously been proposed that long-range RNA interactions between the 3′ and 5′ UTR of coronavirus genomes bring the TRS-L and various TRS-B sites into proximity by folding the ends of the genome together [45], with such interactions observed in TGEV [46,47].

**Figure 5:**
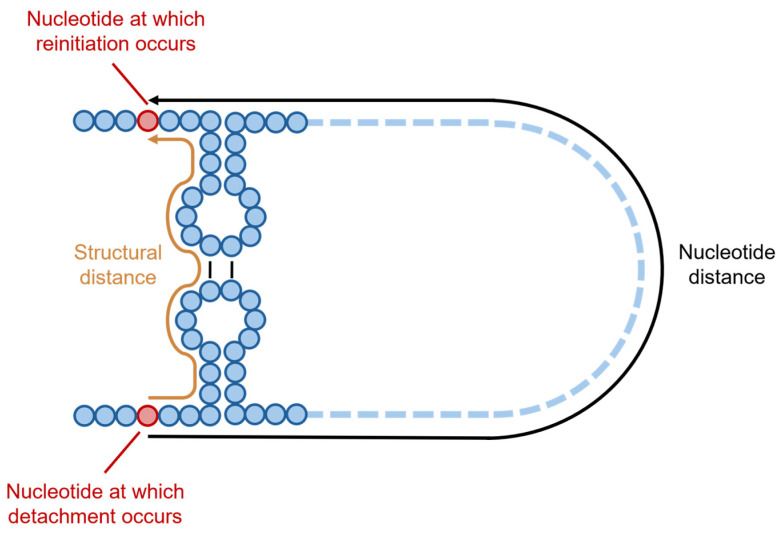
Illustration of the nucleotide distance between two given nucleotides and the structural distance between the same two nucleotides, as defined by Zhou et al. [83]. Whilst nucleotide distance is determined by the number of intervening nucleotides in the primary structure of the genome, moving along only the phosphate backbone of the genome, structural distance is determined by traversing across both the phosphate backbone and base pairs.

When studying the deletion DVGs, Zhou et al. used COMRADES to detect both short-range and long-range interactions within the SARS-CoV-2 genome, correlating the location of pairs of detachment and rejoining with RNA interactions detected through COMRADES [83,94]. A recombination between nucleotides 724 and 29,406 was reported by Zhou et al., with a distance of >28,000 nucleotides observed between these two sites in the genome; however, Zhou et al. also examined the “structural distance” between the two sites of recombination, defining this as “the shortest distance between two nucleotides by traversing the backbone and base pairs” (Figure 5) [83]. The median distance between any two nucleotides in the SARS-CoV-2 genome was observed to be 112 nucleotides, with just under half of all the nucleotides (41%) found within a structural distance of 100 of each other [83].

When examining the structural distance between the pairs of positions observed in recombinant junctions of deletion DVGs in SARS-CoV-2 (such as the recombination between nucleotides 724 and 29,406), the median structural distance was 33, with the overwhelming majority (94%) of deletion events occurring between nucleotides within a structural distance of 100 nucleotides [83]. This finding corroborated the prior suggestion that 3′ and 5′ UTR interactions played a role in directing sgRNA formation [45,51]. A median structural distance of 42 nucleotides was observed between TRS-B sites and the TRS-L site, suggesting that RNA–RNA interactions are bringing these sites into proximity, enabling sgRNA synthesis [83]. During sgRNA formation, the different minimum free energy requirements of the cTRS-B-TRS-L interactions could increase the likelihood of a successful binding between the template and nascent strands, enabling the reinitiation of replication [51]. For example, the cTRS-B sequence found in the partially synthesised negative-sense N sgRNA may more efficiently bind to the TRS-L, promoting more frequent reinitiation of synthesis than the cTRS-B sequence in another partially synthesised sgRNA.

Taken with the finding that DDX1 helicase recruitment is essential for the synthesis of full-length coronavirus gRNA, and that its absence leads to the formation of the shorter sgRNAs during RNA synthesis [49], these findings suggest that RNA secondary structure can encourage the detachment of the viral RTC from the template strand as well as direct the site of reattachment through interactions with other regions of the SAR-CoV-2 genome during sgRNA or DVG synthesis. By using RNAfold to predict the minimum free energy of the region surrounding the two sites in a recombinant junction, it was seen that in both OC43 and MHV the frequency of deletion recombinations in a given region correlated with lower minimum free energy, which suggests that regions commonly seen in recombinant junctions contain more RNA secondary structure [85]. However, the regions with the lowest predicted minimum free energy did not contain much recombination, suggesting that, beyond a certain degree of structural complexity, this link between structure and recombination might not be maintained [85].

When examining the recombinant junctions in SARS-CoV-2, MERS-CoV, and MHV deletion DVGs, it appeared that the two recombining sections of genome had high rates of microhomology around the sites of recombination: more frequent overlaps of 2–7 nucleotides were observed than would be expected if comparing two randomly selected sections of the SARS-CoV-2 or MERS-CoV genomes [86]. The high rates of microhomology between recombining sections of genome have also been reported in flock house virus chimeric recombination events [61]. The small homologous regions shared by the nascent strand, which is held by the viral replication complex, and the prospective template strand on which the replication complex may reinitiate transcription might enable this reattachment by binding the two strands together.

Examining the nucleotide composition around the recombinant junctions showed that in both SARS-CoV-2 and MERS-CoV deletion DVGs, one side of the junction was enriched for uracil but depleted of adenosine and guanine nucleotides relative to the rest of the genome [86]. The other side of the same junction was seen to be enriched for guanine and adenosine but depleted for uracil [86]. SARS-CoV-2 also appeared to show some depletion of cytidine nucleotides in the uracil-enriched side of the junction, whilst MERS-CoV did not [74]. In MHV, uracil enrichment and cytidine depletion was observed on one side of the junction, whilst on the other, adenosine enrichment was seen [86]. However, another study on BCoV and MHV found that adenosine and uracil were enriched on both sides of the junctions present in DVGs [95].

In other viruses, recombinant hotspots appear to cluster in AU-rich regions [96,97,98], with these AU-rich sequences encouraging the slippage of viral replication complexes in part due to weaker base-pairing between the nascent and template strands in the AU-enriched sequences [99]. How the specific nucleotide content of an RNA sequence affects the mechanism of recombination in coronaviruses is yet to be determined.

In addition to elements in the genomic sequence that can influence the sites of recombination, elements of the coronavirus RTC have been implicated in enabling recombination. In MHV, mutation of the nsp14 exonuclease’s catalytic site decreased the overall frequency of deletion DVGs (normalised to the number of reads aligned to the reference genome) as well as the number of unique deletion DVGs detected by ViReMa in comparison to MHV with wild-type nsp14 grown in identical conditions [86]. This suggests that a functional nsp14 increases the rate of RNA recombination in MHV, and whilst not a strict determinant of DVG formation, this exonuclease may play a role in the recombination events that generate DVGs [86]. Examining viral supernatants and cell lysates confirmed that this reduction in DVGs was observable in both settings, and the mutation also reduced the amount of sgRNA that formed during infection, suggesting that nsp14 mutation was directly affecting the ability of the viral replication complex to successfully carry out recombination events rather than affecting the packaging and propagation of the deletion DVGs [86].

This mutation did not affect the rate of microhomology observed between recombining sections of the MHV genome, and this might suggest that the requirements for reattachment of the replication complex are not being altered by this mutation. If the reattachment process was rendered more difficult by this mutation, higher rates of microhomology that might confer stronger binding between the two recombining sections of RNA may be required for the reattachment.

As previously noted, template RNA and nascent strand RNA in the coronavirus RTC is duplexed to a greater degree than other RNA viruses [29], and this duplexed RNA must dissociate in order for the recombination events forming DVGs or sgRNAs to occur. Mismatching of nucleotides during RNA synthesis may enable this dissociation: a mismatched nucleotide on the nascent strand within the active site of the RdRp is not base-paired with the template and instead is “frayed” away from the template strand [100]. This orientates the 3′ end of the nascent strand towards the nucleotide triphosphate entry tunnel of the replication complex and allows nsp13 to engage the free end of the nascent strand, whereupon this helicase enzyme moves in a 5′ to 3′ direction opposite to the RdRp’s direction of travel [100]. As the RdRp backtracks in the opposite direction, the two strands are peeled apart as the nascent strand is fed through the NTP entry tunnel, whilst the template strand is fed back through the main channel that leads to the active site of the RdRp, disassociating the two strands [100].

This dissociation has been suggested as subsequently enabling template-switching and recombination events [100]. In EAV, a mutation in the helicase nsp prevented the production of sgRNA without preventing gRNA formation, demonstrating the importance of the helicase in enabling discontinuous replication in coronaviruses [101]. How the disassociated region of the nascent strand is introduced to a new template is unclear. Given the unidirectional motion of the template gRNA through the replication complex, in order for copybacks and duplication DVGs to form, detachment of the existing template and recruitment of a new template all whilst retaining the nascent strand would need to occur. Without the exchange of template strands or another mechanism of transferring the nascent strand to a new template, the intermolecular recombination events observed in MHV and the recombination events thought to enable the transfer of the HE protein from an avian influenza C virus would not be possible [63,64]. It is also unclear whether the incorporation of mismatched nucleotides during RNA synthesis, and nsp13 mediated backtracking, is the only cause for dissociation of the two strands. As discussed, the presence of RNA secondary structures appears to encourage recombination or template-switching, but whether this is in any way linked to the backtracking of the replication complex is unclear.

Whilst DVG generation may have been considered a random process in the historic literature, recent studies of coronavirus DVGs have led to the observation of hotspots for DVG formation as well as elucidation of the role of RNA secondary structures in determining both the site of RTC detachment and reattachment. This has shown that there are specific mechanisms that might promote the formation of specific DVGs, rather than DVG formation being an entirely random process.

### 6.4. Propagation and Packaging of Coronavirus RNAs

Once they have been formed, DVGs are subject to competition with other DVGs and the gRNA to be replicated and packaged with their fitness at both steps of the lifecycle, determining whether they are propagated or lost from a population. When bound to gRNA, N proteins can interact with other N proteins, leading to the formation of helical viral ribonucleoprotein (vRNP) complexes [102]. The interactions between the N proteins and gRNA are predicted to cause liquid–liquid phase separation (LLPS), forming a “droplet” of condensate within the cytoplasm that contains protein and a single genome [102]. This condensation of protein and a single genome could then proceed to be packaged into a new virion. Due to the high level of sequence conservation between the N proteins of HCoVs and SARS-CoV-2, it is assumed that the SARS-CoV-2 N protein acts in a similar manner to the N proteins of other HCoVs [102].

Constructs consisting of bases 28,273 to 29,533 at the 3′ end of the SARS-CoV-2 genome and the first 1000 bp of the 5′ end of the SARS-CoV-2 genome were shown to bind N proteins and undergo LLPS, with the addition of 0.3kb to 2.4kb of additional nucleotides to the 1000 bp 5′ terminus of the constructs enhancing LLPS [103]. This suggests that, in SARS-CoV-2, the minimal requirements for packaging may be these terminal regions. The preferential packaging of gRNA over sgRNA may be enabled by the loss of the majority of the 5′ N binding regions downstream of the TRS-L sequence.

Whilst it has been suggested that nucleotides 19,674 to 20,340 of the SARS-CoV-2 genome, which contains a portion of the *nsp15* gene, may harbour a packaging signal in SARS-CoV-2 [70], Girgis et al. observed the propagation of DVGs missing this putative packaging signal, suggesting that it is not a strict requirement for packaging [84]. If involved in packaging at all in SARS-CoV-2, this region may instead be an element that enables the preferential packaging of full-length gRNA instead of the more abundant sgRNAs; however, this packaging signal may not be relevant at all to SARS-CoV-2, as it does not appear to be present in any of the lineage A β-coronaviruses [104].

When examining the propagation of SARS-CoV-2 DVGs through multiple passages, only deletion DVGs have been observed as persistently presented across multiple passages [84]. However, the persistence and propagation of other types of DVGs was not examined, as deletion DVGs were the specific focus of this study [84]. Application of the DVGfinder tool to serially passaged OC43 stocks from both BHK-21 and HCT-8 cells, as well as serially passaged MHV in CCL-9.1 cells, revealed that examples of deletion, duplication/insertion, 5′ copyback, and 3′ copyback DVGs could be detected across multiple passages in both viruses, suggesting that all four of these types of DVGs can be packaged and propagated by coronaviruses [85]. The replication and packaging of copybacks is notable, as the 3′ copybacks only contain the 3′ UTR and therefore lack any elements in the 5′ UTR that would enable packaging. Likewise, 5′ copybacks would lack the packaging and replication signals found in the 3′ UTRs. The propagation of these two types of DVGs may suggest that signals in either UTR are dispensable as long as one of the two UTRs is retained. Interestingly, 5′ copybacks were more prevalent than 3′ copybacks in both cell lines, which might imply that two copies of the 5′ replication promoter (one anti-sense and one sense) are more effective than two copies of the 3′ replicative promoter, a phenomenon previously observed in rabies virus [105].

The absence of 5′ UTR elements in the trailer copybacks, and their propagation regardless, might imply that the packaging elements that enable LLPS are not the only route by which RNAs can be packaged by coronaviruses. It is possible that without these elements the packaging efficiency is very low, but the frequency of these DVGs still leads to small numbers making it through this bottleneck. Alternatively, it may be possible that an additional inverted copy of the 3′ UTR can compensate for the loss of the 5′ UTR by providing a site for N binding that promotes LLPS. In comparison to the deletion and insertion/duplication DVGs, smaller numbers of 5′ or 3′ copybacks persisted across serial passages [85]. When examining the proportion of MHV and OC43 DVGs that were persistent, rather than de novo generated at different timepoints during serial passaging, less than 30% of the MHV DVGs of any type were persistent DVGs, with 70% or more of the DVGs at any examined passaged being de novo generated [85]. In contrast, the proportion of OC43 deletion DVGs that were persistent across passages rather than de novo generated reached over 50% during the serial passages and OC43 samples contained more persistent DVGs, but the number of persistent DVGs did vary between the two cell lines that OC43 was grown in [85]. This makes it hard to draw general conclusions about the behaviour of the four types of DVGs across coronaviruses, as their behaviour appears to differ between cell lines as well as between model viruses, but across the viruses and cells lines there tended to be more persistent deletion DVGs than persistent DVGs of any other type. This might suggest that deletion DVGs are better at being propagated than the types.

When comparing NGS datasets produced by the illumina sequencing of either MHV-infected cell monolayers or viral supernatants, and having normalised for the number of aligned reads, higher frequencies of deletion DVGs and sgRNAs were observed in the cell monolayer than in the supernatant, where over five-fold fewer deletion DVGs were detected [86]. This might suggest that the packaging of RNAs that are not the full-length gRNA is a challenging prospect for these RNAs and that only a small portion of the DVGs and sgRNA synthesised within the cellular environment may make it into the extracellular environment to be propagated alongside the wild-type virus. This has also been demonstrated for the deletion DVGs of IAV [106]. Therefore, the packaging efficiency of a DVG may be a critical factor that determines whether it is propagated by the virus during infections.

Unless a unique mechanism of packaging is employed by coronavirus DVGs, it is likely that DVGs are packaged through a similar process as gRNA, in which the first step is the formation of viral nucleoprotein (vRNP) complexes by the binding of N proteins to the genome. Interaction of coronavirus gRNA with the N proteins enhances its recruitment to M proteins, which are embedded in the endoplasmic reticulum–Golgi intermediate compartment (ERGIC), subsequently triggering virion assembly (Figure 2) [107]. The N proteins of SARS-CoV-2, SARS-CoV, and MERS-CoV all display specific binding to a pair of RNA motifs present in their genomes, with the consensus sequences of these two N binding motifs identified as “UCCGCUUGGCC” and “UAAUAGCCGAC” by Fan et al. [108]. Across the three aforementioned HCoVs, these two motifs were distributed across the genome, but the first motif was more abundant in the UTRs, whilst the second motif was more common in the central region of the genomes [108]. Both the N-terminal domain and C-terminal domain, which is also involved in the homodimerisation of N proteins, contain RNA-binding domains [108]. The homodimerisation of the N proteins appears to be required for stable vRNP formation, as the two RNA binding domains in isolation fail to stably bind to RNA [108]. The distribution of the two different N-binding motifs and the importance of N homodimersiation for vRNP formation led Fan et al. to propose that vRNP formation is a two-step process: N protein dimers initially bind to the 3′ and 5′ UTRs of the gRNA, triggering LLPS and a conformational change in the genome that exposes the central region between the UTRs. Subsequently, more N protein can aggregate on the genome and enable the interaction of the N protein-coated gRNA with the other structural proteins [108]. N protein binding to the UTRs appears to drive LLPS specifically [103], whilst N protein binding to the central region promotes solubilisation of the genome and increases the efficiency with which the genome is packaged [109]. By retaining sections of the UTRs, DVGs and sgRNA might be able to bind N proteins and undergo LLPS, but removing the central regions of the genome may limit the amount of N protein that can bind and reduce the packaging efficiency of these RNAs relative to the gRNA.

To reach the site of virion assembly in the ERGIC, the vRNPs exit the DMV replication organelles through the same transmembrane pore that sgRNAs use to enter the cytosol prior to translation (Figure 2) [110]. The LLPS of the vRNPs into a condensate, compared to the uncondensed state of N proteins without gRNA, may enable the translocation of gRNAs out of the replication organelles, but this has not been confirmed. Elements present in the viral RNA promoting LLPS and condensation appear to play a role in the preferential packaging of viral RNAs and the exclusion of non-viral RNAs from the host cell [103]. Where the phosphorylation of the N protein has previously enabled the regulation of sgRNA and gRNA formation, N protein dephosphorylation appears to trigger the transition between RNA synthesis and nucleocapsid assembly [109]. The phosphorylated N protein also appears to suppress the phase separation of the vRNP complexes, whilst in the presence of unmodified N protein, the vRNP more readily undergoes LLPS, initiating further interaction with M proteins [109].

Alongside the M proteins, E proteins are assembled at the ERGIC membrane. Interaction between these proteins with the vRNPs and S proteins incorporates these essential components into the new virions [16,107]. The oligomerisation of the M proteins through interaction with a vRNP complex can induce a curvature in the membrane that is suggested to enable the formation of a viral particle and budding into the ERGIC [107].

Interactions between the structural proteins and vRNP complexes cause the budding of a viral particle into the lumen of the endoplasmic reticulum (ER) and ERGIC, after which β-coronaviruses exploit lysosomes to become exocytosed from the host cell [111]. Initially, the coronaviruses are trafficked through the Golgi apparatus and trans-Golgi network, where the proteins on the viral particles are able to receive post-translational modifications (Figure 2) [112]. After this, β-coronaviruses move into lysosomes and, through exocytic lysosomes, can be released from the host cells without inducing cell lysis [111]. Cryo-EM tomography of SARS-CoV-2-infected Vero cells did find assembled virions in membrane compartments morphologically similar to lysosomes but was not able to definitively show that SARS-CoV-2 used lysosomes to exit the host cells [110]. Alternatively, SARS-CoV-2 may exit cells via small secretory vesicles, harbouring individual particles that can release virions from the cell after trafficking through the ER/ERGIC [113]. Release via these small vesicles was also suggested following cryo-EM tomography studies in SARS-CoV-2-infected Vero cells; however, a key difference between the studies was the timing of the cryo-EM imaging. Eymieux et al., who argue in favour of small vesicle secretion, performed their imaging at ten hours post-infection, as this time point was previously shown as the beginning of SARS-CoV-2 exit [113,114]. In contrast, the study by Mendonça et al., which has been used to suggest that the lysosomal exit pathway may be employed by SARS-CoV-2, studied Vero cells 24 h after infection [110]. The presence of SARS-CoV-2 in lysosomes at the later time point may reflect an attempt by the infected cell to degrade intracellular virions in order to produce peptides for presentation on major histocompatibility complex (MHC) class II receptors [115]. SARS-CoV-2 interacts with the lysosomes, altering their environment by deacidifying the compartments and reducing lysosomal enzyme activity [111]. Therefore, the virus may be actively manipulating lysosomes as part of an immune evasion strategy even if they do not provide an exit mechanism. Having exited the cell, the virus is then free to spread within the host or be transmitted to further hosts, and if spreading to secondary hosts, the DVGs generated during the infection may be passed on with the virus.

## Abbreviations

The following abbreviations are used in this manuscript:

HCoV	Human coronavirus
MERS-CoV	Middle Eastern respiratory syndrome coronavirus
SARS-CoV	Severe acute respiratory syndrome coronavirus
SARS-CoV-2	Severe acute respiratory syndrome 2 coronavirus
CoV	Coronavirus
RTC	Replication/transcription complex
gRNA	Genomic RNA
ORF	Open reading frame
Pp	Polyprotein
Nsp	Non-structural protein
sgRNA	Subgenomic RNA
DMVs	Double-membrane vesicles
DMSs	Double-membrane spherules
CMs	Convoluted membranes
MHV	Mouse hepatitis virus
DMCs	Double-membrane connectors
PL^pro^	Papain-like protease
M^pro^	Main protease
RdRp	RNA-dependent RNA polymerase
UTR	Untranslated region
BCoV	Bovine coronavirus
DVG	Defective viral genome
TRS	Transcriptional regulatory site
TRS-L	Transcriptional regulatory leader site
TRS-B	Transcriptional regulatory body site
TGEV	Transmissible gastroenteritis virus
N	Nucleocapsid
E	Envelope
M	Membrane
S	Spike
HE	Haemagglutinin esterase
HIV-1	Human immunodeficiency virus-1
IBV	Infectious bronchitis virus
NGS	Next-generation sequencing
PHLE	Primary human lung epithelial
IFN	Interferon
IAV	Influenza A virus
MOI	Multiplicity of infection
COMRADES	Crosslinking of matched RNAs and deep sequencing
vRNP	Viral ribonucleoprotein
LLPS	Liquid–liquid phase separation
ERGIC	Endoplasmic reticulum–Golgi intermediate compartment
ER	Endoplasmic reticulum
MHC	Major histocompatibility complex

## References

[1] Walker P.J., Siddell S.G., Lefkowitz E.J., Mushegian A.R., Adriaenssens E.M., Alfenas-Zerbini P., Dempsey D.M., Dutilh B.E., García M.L., Curtis Hendrickson R. (2022). Recent Changes to Virus Taxonomy Ratified by the International Committee on Taxonomy of Viruses. Arch. Virol..

[2] Kesheh M.M., Hosseini P., Soltani S., Zandi M. (2022). An Overview on the Seven Pathogenic Human Coronaviruses. Rev. Med. Virol..

[3] Brucková M., McIntosh K., Kapikian A.Z., Chanock R.M. (1970). The Adaptation of Two Human Coronavirus Strains (OC38 and OC43) to Growth in Cell Monolayers. Proc. Soc. Exp. Biol. Med..

[4] Su S., Wong G., Shi W., Liu J., Lai A.C.K., Zhou J., Liu W., Bi Y., Gao G.F. (2016). Epidemiology, Genetic Recombination, and Pathogenesis of Coronaviruses. Trends Microbiol..

[5] Chen B., Tian E.-K., He B., Tian L., Han R., Wang S., Xiang Q., Zhang S., El Arnaout T., Cheng W. (2020). Overview of Lethal Human Coronaviruses. Signal Transduct. Target. Ther..

[6] Subissi L., Imbert I., Ferron F., Collet A., Coutard B., Decroly E., Canard B. (2014). SARS-CoV ORF1b-Encoded Nonstructural Proteins 12–16: Replicative Enzymes as Antiviral Targets. Antivir. Res..

[7] Nagy P.D., Strating J.R.P.M., van Kuppeveld F.J.M. (2016). Building Viral Replication Organelles: Close Encounters of the Membrane Types. PLoS Pathog..

[8] Snijder E.J., Limpens R.W.A.L., de Wilde A.H., de Jong A.W.M., Zevenhoven-Dobbe J.C., Maier H.J., Faas F.F.G.A., Koster A.J., Bárcena M. (2020). A Unifying Structural and Functional Model of the Coronavirus Replication Organelle: Tracking down RNA Synthesis. PLoS Biol..

[9] Knoops K., Kikkert M., van den Worm S.H.E., Zevenhoven-Dobbe J.C., van der Meer Y., Koster A.J., Mommaas A.M., Snijder E.J. (2008). SARS-Coronavirus Replication Is Supported by a Reticulovesicular Network of Modified Endoplasmic Reticulum. PLoS Biol..

[10] Ulasli M., Verheije M.H., de Haan C.A.M., Reggiori F. (2010). Qualitative and Quantitative Ultrastructural Analysis of the Membrane Rearrangements Induced by Coronavirus. Cell. Microbiol..

[11] de Wilde A.H., Raj V.S., Oudshoorn D., Bestebroer T.M., van Nieuwkoop S., Limpens R.W.A.L., Posthuma C.C., van der Meer Y., Bárcena M., Haagmans B.L. (2013). MERS-Coronavirus Replication Induces Severe in Vitro Cytopathology and is Strongly Inhibited by Cyclosporin A or Interferon-α Treatment. J. Gen. Virol..

[12] Gosert R., Kanjanahaluethai A., Egger D., Bienz K., Baker S.C. (2002). RNA Replication of Mouse Hepatitis Virus Takes Place at Double-Membrane Vesicles. J. Virol..

[13] Wolff G., Limpens R.W.A.L., Zevenhoven-Dobbe J.C., Laugks U., Zheng S., de Jong A.W.M., Koning R.I., Agard D.A., Grünewald K., Koster A.J. (2020). A Molecular Pore Spans the Double Membrane of the Coronavirus Replication Organelle. Science.

[14] Serrano P., Johnson M.A., Almeida M.S., Horst R., Herrmann T., Joseph J.S., Neuman B.W., Subramanian V., Saikatendu K.S., Buchmeier M.J. (2007). Nuclear Magnetic Resonance Structure of the N-Terminal Domain of Nonstructural Protein 3 from the Severe Acute Respiratory Syndrome Coronavirus. J. Virol..

[15] Hurst K.R., Koetzner C.A., Masters P.S. (2013). Characterization of a Critical Interaction between the Coronavirus Nucleocapsid Protein and Nonstructural Protein 3 of the Viral Replicase-Transcriptase Complex. J. Virol..

[16] Klein S., Cortese M., Winter S.L., Wachsmuth-Melm M., Neufeldt C.J., Cerikan B., Stanifer M.L., Boulant S., Bartenschlager R., Chlanda P. (2020). SARS-CoV-2 Structure and Replication Characterized by in Situ Cryo-Electron Tomography. Nat. Commun..

[17] Zimmermann L., Zhao X., Makroczyova J., Wachsmuth-Melm M., Prasad V., Hensel Z., Bartenschlager R., Chlanda P. (2023). SARS-CoV-2 Nsp3 and Nsp4 Are Minimal Constituents of a Pore Spanning Replication Organelle. Nat. Commun..

[18] Huang Y., Wang T., Zhong L., Zhang W., Zhang Y., Yu X., Yuan S., Ni T. (2024). Molecular Architecture of Coronavirus Double-Membrane Vesicle Pore Complex. Nature.

[19] Yang J., Tian B., Wang P., Chen R., Xiao K., Long X., Zheng X., Zhu Y., Sun F., Shi Y. (2025). SARS-CoV-2 NSP3/4 Control Formation of Replication Organelle and Recruitment of RNA Polymerase NSP12. J. Cell Biol..

[20] Ricciardi S., Guarino A.M., Giaquinto L., Polishchuk E.V., Santoro M., Di Tullio G., Wilson C., Panariello F., Soares V.C., Dias S.S.G. (2022). The Role of NSP6 in the Biogenesis of the SARS-CoV-2 Replication Organelle. Nature.

[21] Finkel Y., Mizrahi O., Nachshon A., Weingarten-Gabbay S., Morgenstern D., Yahalom-Ronen Y., Tamir H., Achdout H., Stein D., Israeli O. (2021). The Coding Capacity of SARS-CoV-2. Nature.

[22] Brierley I., Digard P., Inglis S.C. (1989). Characterization of an Efficient Coronavirus Ribosomal Frameshifting Signal: Requirement for an RNA Pseudoknot. Cell.

[23] Ziebuhr J., Snijder E.J., Gorbalenya A.E. (2000). Virus-Encoded Proteinases and Proteolytic Processing in the Nidovirales. J. Gen. Virol..

[24] Ziebuhr J., Thiel V., Gorbalenya A.E. (2001). The Autocatalytic Release of a Putative RNA Virus Transcription Factor from Its Polyprotein Precursor Involves Two Paralogous Papain-like Proteases that Cleave the Same Peptide Bond. J. Biol. Chem..

[25] Malone B., Chen J., Wang Q., Llewellyn E., Choi Y.J., Olinares P.D.B., Cao X., Hernandez C., Eng E.T., Chait B.T. (2021). Structural Basis for Backtracking by the SARS-CoV-2 Replication–Transcription Complex. Proc. Natl. Acad. Sci. USA.

[26] Lv Z., Cano K.E., Jia L., Drag M., Huang T.T., Olsen S.K. (2022). Targeting SARS-CoV-2 Proteases for COVID-19 Antiviral Development. Front. Chem..

[27] Posthuma C.C., te Velthuis A.J.W., Snijder E.J. (2017). Nidovirus RNA Polymerases: Complex Enzymes Handling Exceptional RNA Genomes. Virus Res..

[28] te Velthuis A.J.W., van den Worm S.H.E., Snijder E.J. (2012). The SARS-Coronavirus nsp7+nsp8 Complex is a Unique Multimeric RNA Polymerase Capable of Both de Novo Initiation and Primer Extension. Nucleic Acids Res..

[29] Hillen H.S., Kokic G., Farnung L., Dienemann C., Tegunov D., Cramer P. (2020). Structure of Replicating SARS-CoV-2 Polymerase. Nature.

[30] Pizzato M., Baraldi C., Boscato Sopetto G., Finozzi D., Gentile C., Gentile M.D., Marconi R., Paladino D., Raoss A., Riedmiller I. (2022). SARS-CoV-2 and the Host Cell: A Tale of Interactions. Front. Virol..

[31] Goebel S.J., Hsue B., Dombrowski T.F., Masters P.S. (2004). Characterization of the RNA Components of a Putative Molecular Switch in the 3′ Untranslated Region of the Murine Coronavirus Genome. J. Virol..

[32] Hsue B., Hartshorne T., Masters P.S. (2000). Characterization of an Essential RNA Secondary Structure in the 3′ Untranslated Region of the Murine Coronavirus Genome. J. Virol..

[33] Huang P., Lai M.M.C. (1999). Polypyrimidine Tract-Binding Protein Binds to the Complementary Strand of the Mouse Hepatitis Virus 3′ Untranslated Region, Thereby Altering RNA Conformation. J. Virol..

[34] Williams G.D., Chang R.-Y., Brian D.A. (1999). A Phylogenetically Conserved Hairpin-Type 3′ Untranslated Region Pseudoknot Functions in Coronavirus RNA Replication. J. Virol..

[35] Züst R., Miller T.B., Goebel S.J., Thiel V., Masters P.S. (2008). Genetic Interactions between an Essential 3′ Cis-Acting RNA Pseudoknot, Replicase Gene Products, and the Extreme 3′ End of the Mouse Coronavirus Genome. J. Virol..

[36] Chen S.-C., Olsthoorn R.C.L. (2010). Group-Specific Structural Features of the 5′-Proximal Sequences of Coronavirus Genomic RNAs. Virology.

[37] Miao Z., Tidu A., Eriani G., Martin F. (2021). Secondary Structure of the SARS-CoV-2 5′-UTR. RNA Biol..

[38] Liu P., Li L., Millership J.J., Kang H., Leibowitz J.L., Giedroc D.P. (2007). A U-Turn Motif-Containing Stem-Loop in the Coronavirus 5′ Untranslated Region Plays a Functional Role in Replication. RNA.

[39] Raman S., Bouma P., Williams G.D., Brian D.A. (2003). Stem-Loop III in the 5′ Untranslated Region Is a Cis-Acting Element in Bovine Coronavirus Defective Interfering RNA Replication. J. Virol..

[40] Raman S., Brian D.A. (2005). Stem-Loop IV in the 5′ Untranslated Region Is a Cis-Acting Element in Bovine Coronavirus Defective Interfering RNA Replication. J. Virol..

[41] Escors D., Izeta A., Capiscol C., Enjuanes L. (2003). Transmissible Gastroenteritis Coronavirus Packaging Signal Is Located at the 5′ End of the Virus Genome. J. Virol..

[42] Gorbalenya A.E., Enjuanes L., Ziebuhr J., Snijder E.J. (2006). Nidovirales: Evolving the Largest RNA Virus Genome. Virus Res..

[43] Sola I., Moreno J.L., Zúñiga S., Alonso S., Enjuanes L. (2005). Role of Nucleotides Immediately Flanking the Transcription-Regulating Sequence Core in Coronavirus Subgenomic mRNA Synthesis. J. Virol..

[44] Pasternak A.O., van den Born E., Spaan W.J.M., Snijder E.J. (2001). Sequence Requirements for RNA Strand Transfer during Nidovirus Discontinuous Subgenomic RNA Synthesis. EMBO J..

[45] Sola I., Almazán F., Zúñiga S., Enjuanes L. (2015). Continuous and Discontinuous RNA Synthesis in Coronaviruses. Annu. Rev. Virol..

[46] Moreno J.L., Zúñiga S., Enjuanes L., Sola I. (2008). Identification of a Coronavirus Transcription Enhancer. J. Virol..

[47] Mateos-Gomez P.A., Morales L., Zuñiga S., Enjuanes L., Sola I. (2013). Long-Distance RNA-RNA Interactions in the Coronavirus Genome Form High-Order Structures Promoting Discontinuous RNA Synthesis during Transcription. J. Virol..

[48] Ziv O., Price J., Shalamova L., Kamenova T., Goodfellow I., Weber F., Miska E.A. (2020). The Short- and Long-Range RNA-RNA Interactome of SARS-CoV-2. Mol. Cell.

[49] Wu C.-H., Chen P.-J., Yeh S.-H. (2014). Nucleocapsid Phosphorylation and RNA Helicase DDX1 Recruitment Enables Coronavirus Transition from Discontinuous to Continuous Transcription. Cell Host Microbe.

[50] Ogando N.S., Dalebout T.J., Zevenhoven-Dobbe J.C., Limpens R.W.A.L., van der Meer Y., Caly L., Druce J., de Vries J.J.C., Kikkert M., Bárcena M. (2020). SARS-Coronavirus-2 Replication in Vero E6 Cells: Replication Kinetics, Rapid Adaptation and Cytopathology. J. Gen. Virol..

[51] Wang D., Jiang A., Feng J., Li G., Guo D., Sajid M., Wu K., Zhang Q., Ponty Y., Will S. (2021). The SARS-CoV-2 Subgenome Landscape and its Novel Regulatory Features. Mol. Cell.

[52] de Haan C.A.M., Volders H., Koetzner C.A., Masters P.S., Rottier P.J.M. (2002). Coronaviruses Maintain Viability despite Dramatic Rearrangements of the Strictly Conserved Genome Organization. J. Virol..

[53] de Groot R.J. (2006). Structure, Function and Evolution of the Hemagglutinin-Esterase Proteins of Corona- and Toroviruses. Glycoconj. J..

[54] Viswanathan T., Arya S., Chan S.-H., Qi S., Dai N., Misra A., Park J.-G., Oladunni F., Kovalskyy D., Hromas R.A. (2020). Structural Basis of RNA Cap Modification by SARS-CoV-2. Nat. Commun..

[55] Wu H.-Y., Ke T.-Y., Liao W.-Y., Chang N.-Y. (2013). Regulation of Coronaviral Poly(A) Tail Length during Infection. PLoS ONE.

[56] Brant A.C., Tian W., Majerciak V., Yang W., Zheng Z.-M. (2021). SARS-CoV-2: From its Discovery to Genome Structure, Transcription, and Replication. Cell Biosci..

[57] Tvarogová J., Madhugiri R., Bylapudi G., Ferguson L.J., Karl N., Ziebuhr J. (2019). Identification and Characterization of a Human Coronavirus 229E Nonstructural Protein 8-Associated RNA 3′-Terminal Adenylyltransferase Activity. J. Virol..

[58] Peng Y.-H., Lin C.-H., Lin C.-N., Lo C.-Y., Tsai T.-L., Wu H.-Y. (2016). Characterization of the Role of Hexamer AGUAAA and Poly(A) Tail in Coronavirus Polyadenylation. PLoS ONE.

[59] Dimmock N.J., Easton A.J. (2014). Defective Interfering Influenza Virus RNAs: Time to Reevaluate Their Clinical Potential as Broad-Spectrum Antivirals?. J. Virol..

[60] Genoyer E., López C.B. (2019). The Impact of Defective Viruses on Infection and Immunity. Annu. Rev. Virol..

[61] Peccoud J., Lequime S., Moltini-Conclois I., Giraud I., Lambrechts L., Gilbert C. (2018). A Survey of Virus Recombination Uncovers Canonical Features of Artificial Chimeras Generated During Deep Sequencing Library Preparation. G3 Genes/Genomes/Genet..

[62] Duhaut S.D., Dimmock N.J. (1998). Heterologous Protection of Mice from a Lethal Human H1N1 Influenza A Virus Infection by H3N8 Equine Defective Interfering Virus: Comparison of Defective RNA Sequences Isolated from the DI Inoculum and Mouse Lung. Virology.

[63] Zhang X., Kousoulas K.G., Storz J. (1992). The Hemagglutinin/Esterase Gene of Human Coronavirus Strain OC43: Phylogenetic Relationships to Bovine and Murine Coronaviruses and Influenza C Virus. Virology.

[64] Luytjes W., Bredenbeek P.J., Noten A.F., Horzinek M.C., Spaan W.J. (1988). Sequence of Mouse Hepatitis Virus A59 mRNA 2: Indications for RNA Recombination between Coronaviruses and Influenza C Virus. Virology.

[65] Mahmoud M., Gobet N., Cruz-Dávalos D.I., Mounier N., Dessimoz C., Sedlazeck F.J. (2019). Structural Variant Calling: The Long and the Short of it. Genome Biol..

[66] Sapoval N., Mahmoud M., Jochum M.D., Liu Y., Elworth R.A.L., Wang Q., Albin D., Ogilvie H.A., Lee M.D., Villapol S. (2021). SARS-CoV-2 Genomic Diversity and the Implications for qRT-PCR Diagnostics and Transmission. Genome Res..

[67] Chaturvedi S., Vasen G., Pablo M., Chen X., Beutler N., Kumar A., Tanner E., Illouz S., Rahgoshay D., Burnett J. (2021). Identification of a Therapeutic Interfering Particle-A Single-Dose SARS-CoV-2 Antiviral Intervention with a High Barrier to Resistance. Cell.

[68] Chaturvedi S., Beutler N., Vasen G., Pablo M., Chen X., Calia G., Buie L., Rodick R., Smith D., Rogers T. (2022). A Single-Administration Therapeutic Interfering Particle Reduces SARS-CoV-2 Viral Shedding and Pathogenesis in Hamsters. Proc. Natl. Acad. Sci. USA.

[69] Xiao Y., Lidsky P.V., Shirogane Y., Aviner R., Wu C.-T., Li W., Zheng W., Talbot D., Catching A., Doitsh G. (2021). A Defective Viral Genome Strategy Elicits Broad Protective Immunity against Respiratory Viruses. Cell.

[70] Yao S., Narayanan A., Majowicz S.A., Jose J., Archetti M. (2021). A Synthetic Defective Interfering SARS-CoV-2. PeerJ.

[71] Routh A., Johnson J.E. (2014). Discovery of Functional Genomic Motifs in Viruses with ViReMa–A Virus Recombination Mapper–for Analysis of next-Generation Sequencing Data. Nucleic Acids Res..

[72] Sotcheff S., Zhou Y., Yeung J., Sun Y., Johnson J.E., Torbett B.E., Routh A.L. (2023). ViReMa: A Virus Recombination Mapper of next-Generation Sequencing Data Characterizes Diverse Recombinant Viral Nucleic Acids. GigaScience.

[73] Beauclair G., Mura M., Combredet C., Tangy F., Jouvenet N., Komarova A.V. (2018). DI-Tector: Defective Interfering Viral Genomes’ Detector for next-Generation Sequencing Data. RNA.

[74] Sun Y., Kim E.J., Felt S.A., Taylor L.J., Agarwal D., Grant G.R., López C.B. (2019). A Specific Sequence in the Genome of Respiratory Syncytial Virus Regulates the Generation of Copy-Back Defective Viral Genomes. PLoS Pathog..

[75] Achouri E., Felt S.A., Hackbart M., Rivera-Espinal N.S., López C.B. (2023). VODKA2: A Fast and Accurate Method to Detect Non-Standard Viral Genomes from Large RNA-Seq Datasets. RNA.

[76] Boussier J., Munier S., Achouri E., Meyer B., Crescenzo-Chaigne B., Behillil S., Enouf V., Vignuzzi M., van der Werf S., Naffakh N. (2020). RNA-Seq Accuracy and Reproducibility for the Mapping and Quantification of Influenza Defective Viral Genomes. RNA.

[77] Olmo-Uceda M.J., Muñoz-Sánchez J.C., Lasso-Giraldo W., Arnau V., Díaz-Villanueva W., Elena S.F. (2022). DVGfinder: A Metasearch Tool for Identifying Defective Viral Genomes in RNA-Seq Data. Viruses.

[78] Snijder E.J., den Boon J.A., Horzinek M.C., Spaan W.J. (1991). Characterization of Defective Interfering RNAs of Berne Virus. J. Gen. Virol..

[79] Hofmann M.A., Sethna P.B., Brian D.A. (1990). Bovine Coronavirus mRNA Replication Continues throughout Persistent Infection in Cell Culture. J. Virol..

[80] Pénzes Z., Wroe C., Brown T.D., Britton P., Cavanagh D. (1996). Replication and Packaging of Coronavirus Infectious Bronchitis Virus Defective RNAs Lacking a Long Open Reading Frame. J. Virol..

[81] van der Most R.G., Bredenbeek P.J., Spaan W.J. (1991). A Domain at the 3′ End of the Polymerase Gene Is Essential for Encapsidation of Coronavirus Defective Interfering RNAs. J. Virol..

[82] Méndez A., Smerdou C., Izeta A., Gebauer F., Enjuanes L. (1996). Molecular Characterization of Transmissible Gastroenteritis Coronavirus Defective Interfering Genomes: Packaging and Heterogeneity. Virology.

[83] Zhou T., Gilliam N.J., Li S., Spandau S., Osborn R.M., Connor S., Anderson C.S., Mariani T.J., Thakar J., Dewhurst S. (2023). Generation and Functional Analysis of Defective Viral Genomes during SARS-CoV-2 Infection. mBio.

[84] Girgis S., Xu Z., Oikonomopoulos S., Fedorova A.D., Tchesnokov E.P., Gordon C.J., Schmeing T.M., Götte M., Sonenberg N., Baranov P.V. (2022). Evolution of Naturally Arising SARS-CoV-2 Defective Interfering Particles. Commun. Biol..

[85] Hillung J., Olmo-Uceda M.J., Muñoz-Sánchez J.C., Elena S.F. (2024). Accumulation Dynamics of Defective Genomes during Experimental Evolution of Two Betacoronaviruses. Viruses.

[86] Gribble J., Stevens L.J., Agostini M.L., Anderson-Daniels J., Chappell J.D., Lu X., Pruijssers A.J., Routh A.L., Denison M.R. (2021). The Coronavirus Proofreading Exoribonuclease Mediates Extensive Viral Recombination. PLoS Pathog..

[87] Molenkamp R., Rozier B.C.D., Greve S., Spaan W.J.M., Snijder E.J. (2000). Isolation and Characterization of an Arterivirus Defective Interfering RNA Genome. J. Virol..

[88] Lamers M.M., Haagmans B.L. (2022). SARS-CoV-2 Pathogenesis. Nat. Rev. Microbiol..

[89] Ziegler C.G.K., Miao V.N., Owings A.H., Navia A.W., Tang Y., Bromley J.D., Lotfy P., Sloan M., Laird H., Williams H.B. (2021). Impaired Local Intrinsic Immunity to SARS-CoV-2 Infection in Severe COVID-19. Cell.

[90] Cave D.R., Hendrickson F.M., Huang A.S. (1985). Defective Interfering Virus Particles Modulate Virulence. J. Virol..

[91] Frensing T., Heldt F.S., Pflugmacher A., Behrendt I., Jordan I., Flockerzi D., Genzel Y., Reichl U. (2013). Continuous Influenza Virus Production in Cell Culture Shows a Periodic Accumulation of Defective Interfering Particles. PLoS ONE.

[92] Lin Y.J., Zhang X., Wu R.C., Lai M.M. (1996). The 3′ Untranslated Region of Coronavirus RNA Is Required for Subgenomic mRNA Transcription from a Defective Interfering RNA. J. Virol..

[93] Ranum J.N., Ledwith M.P., Alnaji F.G., Diefenbacher M., Orton R., Sloan E., Güereca M., Feltman E.M., Smollett K., da Silva Filipe A. (2024). Cryptic Proteins Translated from Deletion-Containing Viral Genomes Dramatically Expand the Influenza Virus Proteome. Nucleic Acids Res..

[94] Ziv O., Gabryelska M.M., Lun A.T.L., Gebert L.F.R., Sheu-Gruttadauria J., Meredith L.W., Liu Z.-Y., Kwok C.K., Qin C.-F., Macrae I.J. (2018). COMRADES Determines in Vivo RNA Structures and Interactions. Nat. Methods.

[95] Lin C.-H., Chen B., Chao D.-Y., Hsieh F.-C., Yang C.-C., Hsu H.-W., Tam H.-M.-H., Wu H.-Y. (2023). Unveiling the Biology of Defective Viral Genomes in Vitro and in Vivo: Implications for Gene Expression and Pathogenesis of Coronavirus. Virol. J..

[96] Shapka N., Nagy P.D. (2004). The AU-Rich RNA Recombination Hot Spot Sequence of Brome Mosaic Virus is Functional in Tombusviruses: Implications for the Mechanism of RNA Recombination. J. Virol..

[97] Vives M.C., Rubio L., Sambade A., Mirkov T.E., Moreno P., Guerri J. (2005). Evidence of Multiple Recombination Events between Two RNA Sequence Variants within a Citrus tristeza virus Isolate. Virology.

[98] Ohshima K., Tomitaka Y., Wood J.T., Minematsu Y., Kajiyama H., Tomimura K., Gibbs A.J. (2007). Patterns of Recombination in Turnip Mosaic Virus Genomic Sequences Indicate Hotspots of Recombination. J. Gen. Virol..

[99] Barr J.N., Wertz G.W. (2001). Polymerase Slippage at Vesicular Stomatitis Virus Gene Junctions To Generate Poly(A) Is Regulated by the Upstream 3′-AUAC-5′ Tetranucleotide: Implications for the Mechanism of Transcription Termination. J. Virol..

[100] Malone B., Urakova N., Snijder E.J., Campbell E.A. (2022). Structures and Functions of Coronavirus Replication–Transcription Complexes and Their Relevance for SARS-CoV-2 Drug Design. Nat. Rev. Mol. Cell Biol..

[101] van Marle G., van Dinten L.C., Spaan W.J.M., Luytjes W., Snijder E.J. (1999). Characterization of an Equine Arteritis Virus Replicase Mutant Defective in Subgenomic mRNA Synthesis. J. Virol..

[102] Cubuk J., Alston J.J., Incicco J.J., Singh S., Stuchell-Brereton M.D., Ward M.D., Zimmerman M.I., Vithani N., Griffith D., Wagoner J.A. (2021). The SARS-CoV-2 Nucleocapsid Protein is Dynamic, Disordered, and Phase Separates with RNA. Nat. Commun..

[103] Iserman C., Roden C.A., Boerneke M.A., Sealfon R.S.G., McLaughlin G.A., Jungreis I., Fritch E.J., Hou Y.J., Ekena J., Weidmann C.A. (2020). Genomic RNA Elements Drive Phase Separation of the SARS-CoV-2 Nucleocapsid. Mol. Cell.

[104] Masters P.S. (2019). Coronavirus Genomic RNA Packaging. Virology.

[105] Finke S., Conzelmann K.-K. (1999). Virus Promoters Determine Interference by Defective RNAs: Selective Amplification of Mini-RNA Vectors and Rescue from cDNA by a 3′ Copy-Back Ambisense Rabies Virus. J. Virol..

[106] Alnaji F.G., Reiser W.K., Rivera-Cardona J., Te Velthuis A.J.W., Brooke C.B. (2021). Influenza A Virus Defective Viral Genomes Are Inefficiently Packaged into Virions Relative to Wild-Type Genomic RNAs. mBio.

[107] Zhang Z., Nomura N., Muramoto Y., Ekimoto T., Uemura T., Liu K., Yui M., Kono N., Aoki J., Ikeguchi M. (2022). Structure of SARS-CoV-2 Membrane Protein Essential for Virus Assembly. Nat. Commun..

[108] Fan S., Sun W., Fan L., Wu N., Sun W., Ma H., Chen S., Li Z., Li Y., Zhang J. (2022). The Highly Conserved RNA-Binding Specificity of Nucleocapsid Protein Facilitates the Identification of Drugs with Broad Anti-Coronavirus Activity. Comput. Struct. Biotechnol. J..

[109] Carlson C.R., Asfaha J.B., Ghent C.M., Howard C.J., Hartooni N., Safari M., Frankel A.D., Morgan D.O. (2020). Phosphoregulation of Phase Separation by the SARS-CoV-2N Protein Suggests a Biophysical Basis for Its Dual Functions. Mol. Cell.

[110] Mendonça L., Howe A., Gilchrist J.B., Sheng Y., Sun D., Knight M.L., Zanetti-Domingues L.C., Bateman B., Krebs A.-S., Chen L. (2021). Correlative Multi-Scale Cryo-Imaging Unveils SARS-CoV-2 Assembly and Egress. Nat. Commun..

[111] Ghosh S., Dellibovi-Ragheb T.A., Kerviel A., Pak E., Qiu Q., Fisher M., Takvorian P.M., Bleck C., Hsu V.W., Fehr A.R. (2020). β-Coronaviruses Use Lysosomes for Egress Instead of the Biosynthetic Secretory Pathway. Cell.

[112] Fung T.S., Liu D.X. (2018). Post-Translational Modifications of Coronavirus Proteins: Roles and Function. Future Virol..

[113] Eymieux S., Uzbekov R., Rouillé Y., Blanchard E., Hourioux C., Dubuisson J., Belouzard S., Roingeard P. (2021). Secretory Vesicles are the Principal Means of SARS-CoV-2 Egress. Cells.

[114] Eymieux S., Rouillé Y., Terrier O., Seron K., Blanchard E., Rosa-Calatrava M., Dubuisson J., Belouzard S., Roingeard P. (2021). Ultrastructural Modifications Induced by SARS-CoV-2 in Vero Cells: A Kinetic Analysis of Viral Factory Formation, Viral Particle Morphogenesis and Virion Release. Cell. Mol. Life Sci. CMLS.

[115] Watts C. (2012). The Endosome–Lysosome Pathway and Information Generation in the Immune System. Biochim. Biophys. Acta.

